# Enhanced Optical Properties of ZnO and CeO_2_-coated ZnO Nanostructures Achieved Via Spherical Nanoshells Growth On A Polystyrene Template

**DOI:** 10.1038/s41598-017-03905-4

**Published:** 2017-06-16

**Authors:** Asmaa Eltayeb, Stephen Daniels, Enda McGlynn

**Affiliations:** 1 School of Electronic Engineering, National Centre for Plasma Science and Technology, Dublin City University, Glasnevin, Dublin 9, Ireland; 2 School of Physical Sciences, National Centre for Plasma Science and Technology, Dublin City University, Glasnevin, Dublin 9, Ireland

## Abstract

In this paper, ZnO, CeO_2_ and CeO_2_-coated ZnO nanostructures were synthesised by simple and efficient low temperature wet chemical methods on Si (100) and quartz substrates. The ZnO films were prepared by a drop coating deposition method. This was then combined with a thin layer of the redox active material CeO_2_ to form CeO_2_-coated ZnO films. Spherical ZnO nanoshell structures and CeO_2_-coated ZnO nanoshells have been prepared using polystyrene (PS) sphere monolayer templates. The structural properties and morphologies of the nanostructures were analysed by x-ray diffraction (XRD) and scanning electron microscopy (SEM). The nanostructure compositions are studied in more detail using secondary ion mass spectroscopy (SIMS). The optical properties of the nanostructures were measured using ultraviolet-visible (UV-Vis) absorption spectroscopy in order to ascertain the effects of the nanoshell structures and the whispering gallery modes associated with these structures on the optical properties of the deposits. Our data show UV and visible light absorption was very significantly enhanced due to this nanostructuring.

## Introduction

Enhanced broadband light absorption by nanostructuring of materials, recently described by Yao *et al*.^1^., is considered an important new engineering design parameter for high performance solar cells and protectors. This new method of light management uses low quality factor whispering gallery resonant modes inside spherical nanoshell structures where the geometry of the structure dramatically improves the absorption and reduces adverse directionality effects due to the substantial enhancement of the effective light path in the active material^2, 3^. Applying this important recent development in conjunction with a redox mediator and a UV absorber, such as CeO_2_ and ZnO^4, 5^, can result in enhancement in the UV light absorption and optical properties, which can in turn be used for more effective applications in a variety of technologies, especially given the relatively favourable band alignment for charge transfer, with a likely small contact potential (see Supplementary Figure I).

Materials based on CeO_2_ are extensively used in many applications including oxygen ion conduction in solid oxide fuel cells^6^, UV absorption^5, 7^, two step thermochemical cycling^8^ and gas sensing^9^. Apart from the well-known oxygen storage capacity of CeO_2_ and its redox properties, CeO_2_ is also suitable for many personal care products specifically related to its ability to block ultraviolet radiation^10–12^. Similarly, ZnO is an important and promising material with many potential applications in short-wavelength optoelectronic and other devices^13^, such as transparent conductive films, surface electro-acoustic wave devices, ultraviolet emitters, cold cathode emitters etc. It has a wide and direct bandgap energy of 3.3 eV at room temperature^14^ and many useful properties such as transparency in the visible range, electrochemical stability and non-toxicity^15, 16^. Therefore, combining both materials (ZnO and CeO_2_) can result in a material with enhanced/unique UV absorption and high stability at high temperature and high material hardness^5^.

ZnO nanostructures have recently attracted a great attention due to their interesting properties for photonic applications and the variety of morphological structures which can be achieved including nanorods^17^, nanorings^18^, nanowires^19^ and nanobelts^20^. Among these various structures, hollow nanostructures are interesting structures for applications such as photocatalysis^21^, solar cells^22^, drug delivery^23^ and much more^24–26^. Synthesising hollow ZnO nanostructures has been recently done using various templates. For example, Sun *et al*.^27–29^ report extraordinary visible-light responses for ZnO hollow microsphere structures synthesised by their recently developed two-step self-assembly concept. These interesting studies were inspired by examples from the biological realm, including the fly compound-eye and fish-scale structures. Jiang *et al*. synthesised ZnO hollow spheres using ethanol droplets as soft templates while Neves *et al*. synthesised ZnO hollow spheres by coating polystyrene beads. Others, like Li *et al*.^30^, Shen *et al*.^31^ and Deng *et al*.^32^, synthesised ZnO hollow particles using a template-free solution method, template-free evaporation method and template-free sonochemical fabrication method, respectively. Although these proposed methods are described as simple and inexpensive, the drop coating method (proposed by Byrne *et al*.^17, 33^) using a template of polystyrene beads, yields samples with a patterned spherical nanoshells of ZnO with improved crystallinity, purity and optical properties.

**Figure 1:**
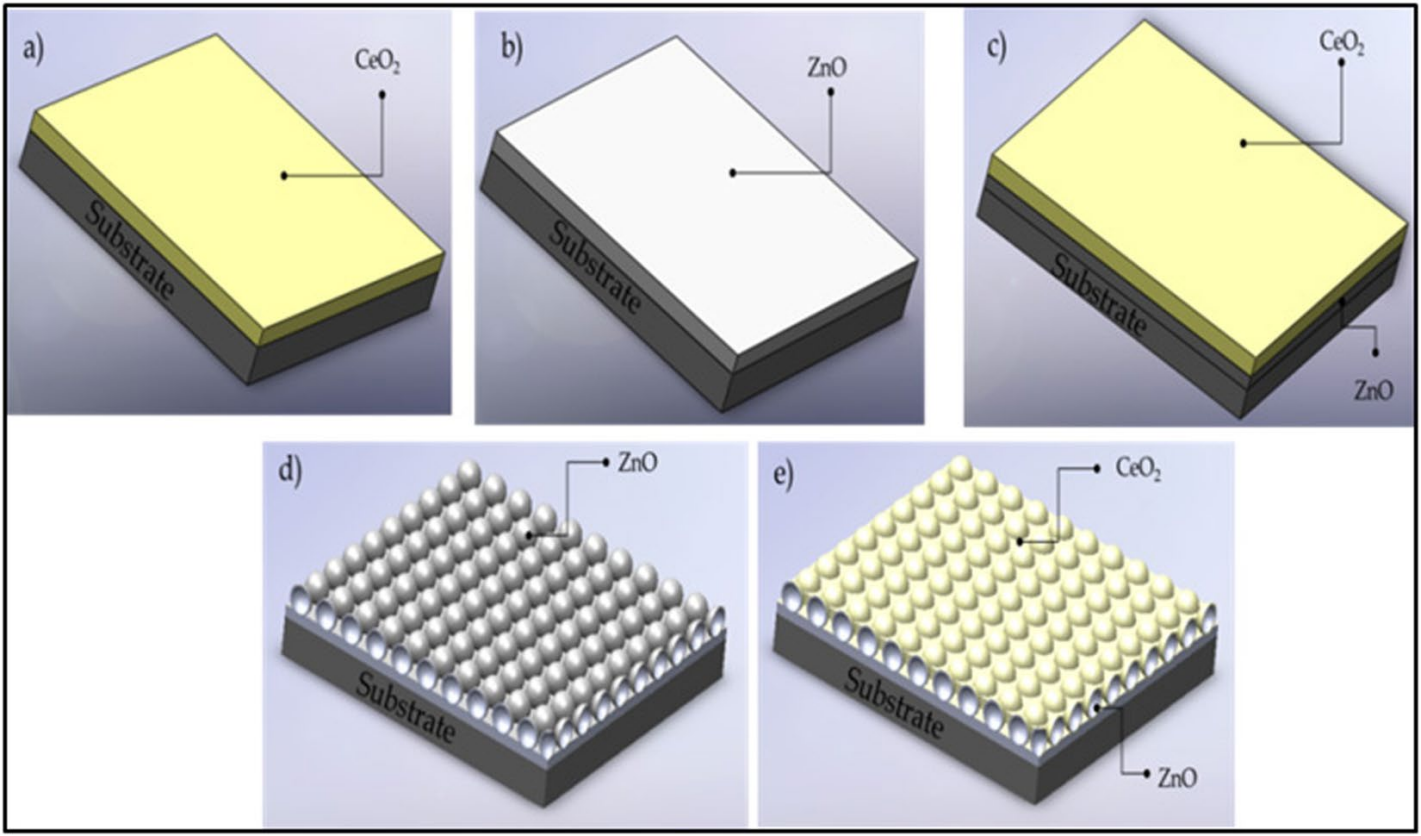
Morphologies used in this study: (**a**) CeO_2_, (**b**) ZnO and (**c**) CeO_2_-coated ZnO films, and; (**d**) ZnO and (**e**) CeO_2_-coated ZnO spherical nanoshells. All samples were deposited on both Si (100) and quartz substrates.

The aim of the present study is to engineer patterned spherical nanoshells of ZnO coated with a CeO_2_ film to achieve enhanced optical properties (UV absorption) primarily for solar-thermal-related applications. The samples used in this study are shown in Fig. 1. The ZnO-CeO_2_ nanostructures were synthesised by drop coating and pulsed DC magnetron sputtering and characterised by XRD, SEM, SIMS and UV-Vis spectroscopy. To the best of our knowledge, using drop coating of PS sphere templates to engineer patterned spherical nanoshells of ZnO has not been reported previously, and is a very simple and versatile method. Our work provides useful information on the influence of the nanoshell geometry on the absorption properties of various combinations of these two types of materials. The addition of the CeO_2_ thin film on top of the ZnO hollow nanostructured deposit enhances the UV light absorption further and provides additional functionality such as oxygen storage capacity via changes in stoichiometry. The redox properties of these types of CeO_2_ films grown in our group were reported in ref. 34.

## Experimental

### Material Synthesis

Prior to deposition, Si(100) and quartz substrates were cleaved to the desired size (2 × 2 cm) and the substrates were ultrasonically cleaned using acetone and a decontamination solution (30905 Aldrich) and then rinsed with deionised water and blown dry with a nitrogen stream. The ZnO layers used to generate ZnO films were prepared by a method initially proposed and demonstrated by Greene *et al*.^35, 36^ and further developed by Byrne *et al*.^17, 33, 37^. This method involves drop coating a mixture of zinc acetate (5 mM) in anhydrous ethanol solution onto the substrate surface for a period of 20–25 seconds before being rinsed with fresh ethanol and dried with a nitrogen stream. This process was repeated approximately 60 times (1 time yields approximately a film thickness of 2 nm). The substrates were then annealed at 350 °C for 20 minutes, yielding a uniform textured nanocrystalline ZnO film with a film thickness of 120 ± 10 nm. Patterned spherical ZnO nanoshells were fabricated on a PS sphere template using the same deposition method for the ZnO films. A monolayer of the PS spheres with a diameter of ca. 600 nm (solid content of ~10 wt.%, Fisher Scientific) were generated by a self-assembly process on the surface of DI water, at room temperature, and then transferred onto bare substrates^33^. The deposited close-packed PS sphere monolayer was then heated at 90 °C for 30 seconds to cause them to adhere better to the substrate surface without significantly deforming the spheres. The PS spheres were then O_2_ plasma treated (Oxford Instruments Plasmalab 80Plus) at a power of 300 W, a pressure of 100 mbar, an oxygen flow rate of 50 sccm for 25 seconds to reduce the sphere diameter from 600 nm to ~520 nm allowing enough space between the spheres for a connected and mechanically stable ZnO nanoshell structure post-deposition. The nanostructured CeO_2_ films are prepared on previously cleaned substrates by pulsed DC magnetron sputtering using the same deposition procedures to the ones described in refs 34 and  38. The process is repeated three times to yield a uniform CeO_2_ thickness of 120 ± 10 nm.

For the CeO_2_-coated ZnO films, the substrate is coated with approximately 40 layers of the 5 mM zinc acetate solution to yield a uniform ZnO film thickness of 80 ± 10 nm. The sample is then transferred to the sputtering chamber to deposit an approximately 50 ± 10 nm of nanostructured CeO_2_ film on top of the ZnO. This resulted in a 120 ± 10 nm thick CeO_2_-coated ZnO films to allow for a straight comparison study. The same process was repeated to fabricate the CeO_2_-coated ZnO spherical nanoshells. A process flow chart is provided in Supplementary Figure II. Some CeO_2_ films were also grown without ZnO, for the purposes of optical studies as mentioned below. These latter nanostructured CeO_2_ films are prepared on previously cleaned substrates by pulsed DC magnetron sputtering using the same deposition procedures to the ones described in refs 34, 38. The process is repeated three times to yield a uniform CeO_2_ thickness of 120 ± 10 nm. Table 1 summaries the sample structures used in this study and their associated labels.

**Table 1:** Associated labels for sample structures used in this study.

Sample Label	Sample Structure Description
C_F	CeO_2_ Films
Z_F	ZnO Films
C_Z_F	CeO_2_-coated ZnO Films
Z_NS	ZnO Nanoshells
C_Z_NS	CeO_2_-coated ZnO Nanoshells

### Characterisation

The structural properties of the pure and CeO_2_-coated ZnO samples were measured using a Bruker D8 Advance X-ray Diffractometer system with CuK_α_ radiation of wavelength λ = 1.5418 Å. The XRD measurements were carried out in locked coupled mode in a 2θ range from 20° to 60°. Sample morphology was studied using SEM (Karl-Zeiss EVO series and Hitachi S-5500 field emission (FE) SEM). The sample composition was studied using SIMS (Millbrook MiniSIMS Alpha). The optical absorption properties of the samples were studied at room temperature (RT) using a Perkin Elmer Lambda 40 UV-Vis spectrometer in the range from 200 to 800 nm with a resolution of 4 nm.

## Results And Discussions

### XRD Measurements

XRD patterns of the ZnO film (Z_F) and ZnO nanoshell (Z_NS); and, the CeO_2_-coated ZnO film (C_Z_F) and CeO_2_-coated ZnO nanoshell (C_Z_NS), as-deposited and annealed at 500 °C and 800 °C in air for 30 minutes are shown in Supplementary Figures III and IV, respectively. The XRD scans of all the Z_F, Z_NS, C_Z_F and C_Z_NS show that the crystalline quality of the samples is systematically improved as a result of annealing. The reflected x-ray intensity and reflection peak full width at half maximum (FWHM) of the CeO_2_ (111) and ZnO (002) XRD peaks are used as an indicator of the crystallinity quality of the CeO_2_ and ZnO deposits. The FWHM data is provided in Supplementary Table I and a detailed account of the XRD measurements is given in Supplementary section S1.

### SEM Measurements

**Figure 2:**
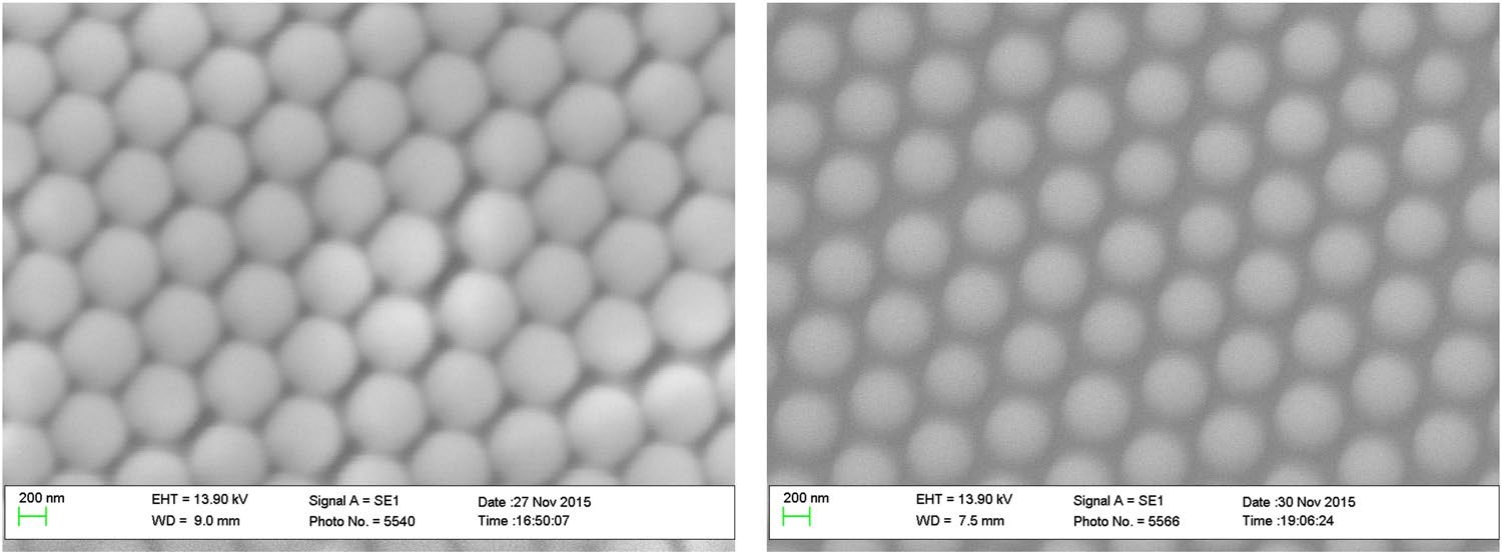
SEM images of the polystyrene sphere monolayer deposited on a Si (100) substrate before (left) and after (right) O_2_ plasma treatment. The sphere diameters reduced from 600 nm to 520 nm when exposed to an O_2_ plasma for 25 seconds.

Figure 2 shows SEM images of the PS sphere monolayer template, before and after the O_2_ plasma treatment. As shown in Fig. 2(left), a PS sphere monolayer is observed on a Si (100) substrate without aggregation or multiple layer accumulation. Figure 2(right) clearly shows a reduction in the sphere diameters (from 600 nm to ~520 nm) after the O_2_ plasma treatment, allowing enough space around the spheres for the Z_NS structures to fully interconnect during growth to ensure mechanical stability. There are some examples of slight movements of spheres, or of a sphere detaching, during the etch despite prior heating at 110 °C for 30 seconds to ensure good adherence, but these are very occasional and do not compromise the overall nanostructure integrity.

**Figure 3:**
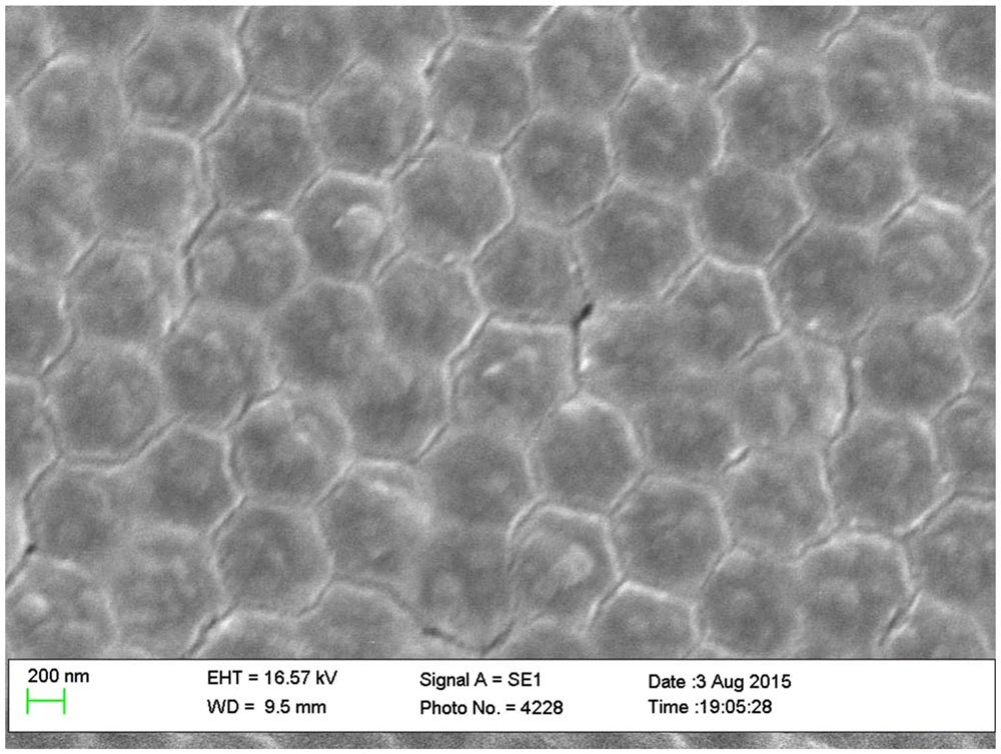
Plan view SEM image of the spherical Z_NS before complete removal of PS nanospheres i.e. samples were simply heated at 350 °C for 30 minutes to decompose the zinc acetate into zinc oxide.

**Figure 4:**
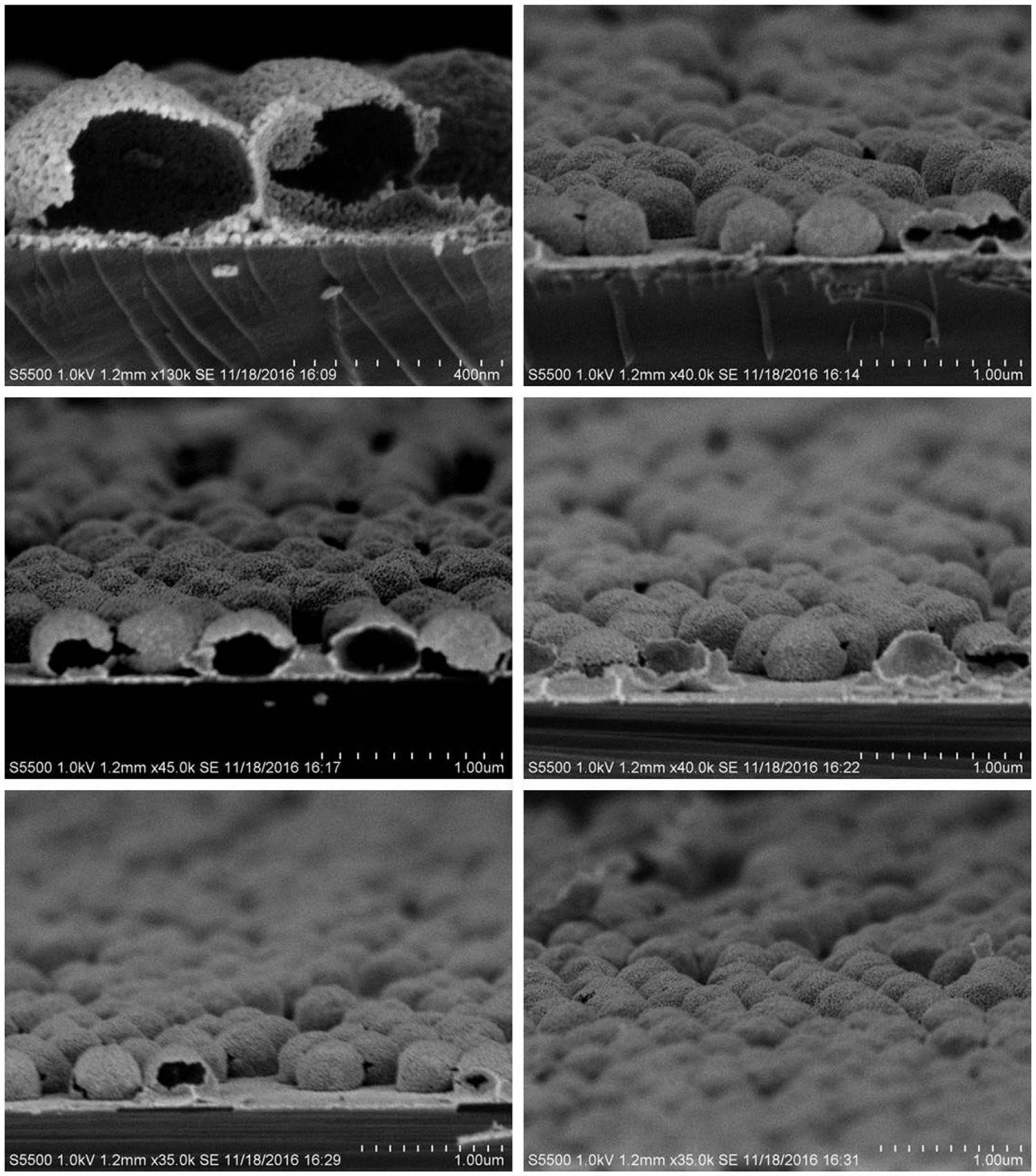
FE-SEM images of the spherical nanoshell structures after the removal of the PS spheres (i.e. annealed at 500 °C for 30 minutes in air). The samples are deposited on both Si (100) and quartz substrates. The SEM images show 90° view of the Z_NS structures at different positions, with clear evidence of internal voids following PS sphere removal.

After the deposition of the zinc acetate films on the PS spheres, the structural morphology of the ZnO deposits depended on the post-deposition annealing temperature. Figure 3 shows a plan view of the zinc acetate decomposed into ZnO nanostructures by annealing the sample at 350 °C for 30 minutes. Spherical nano core-shell structures consisting of the PS sphere core and ZnO shells with a total diameter of ca.~60  nm are formed, as shown in Fig. 4. The thickness of the ZnO is estimated to be ~80 nm on the CeO_2_-coated ZnO sample, as shown in the cross sectional view of the fractured nanostructures. Other views of the spherical nanostructures, after a complete removal of the PS spheres by carbonisation (i.e. by annealing at 500 °C for 30 minutes in air) and the addition of the thin CeO_2_ film (~50 ± 10 nm thick), are also shown in Fig. 4.

### Chemical composition measurements

Information on the chemical composition and impurity content in the samples were obtained by making SIMS measurements at different locations throughout the deposit. Supplementary Figure V shows the SIMS spectra of the C_Z_F in the mass region from 60 to 200 amu, at the boundary where the two materials meet. Sputtered CeO_2_ SIMS spectra showed secondary ion peaks of Ce^+^, CeO^+^ and CeO_2_^+ ^^34^. As more scans are performed and the probing depth increased due to surface sputtering by the Ga ion beam, Zn^+^ and ZnO^+^ peaks start to appear and their intensity increased with the increase in the number of scans. Three different Zn isotopes are observed for the Zn ions, ^64^Zn, ^66^Zn and ^68^Zn. These SIMS data clearly shows evidence of an abrupt interface between the ZnO and CeO_2_ materials. Supplementary Figure VI presents the SIMS depth profiling data of the relative secondary ion emission yields (^64^Zn^+^, Ce^+^, CeO^+^ and CeO_2_^+^) as a function of depth at the boundary of the CeO_2_-coated ZnO composite layers grown on Si(100) substrate. Further details on the SIMS measurements are provided in Supplementary section S2.

### UV-Vis optical absorbance and directionality measurements

**Figure 5:**
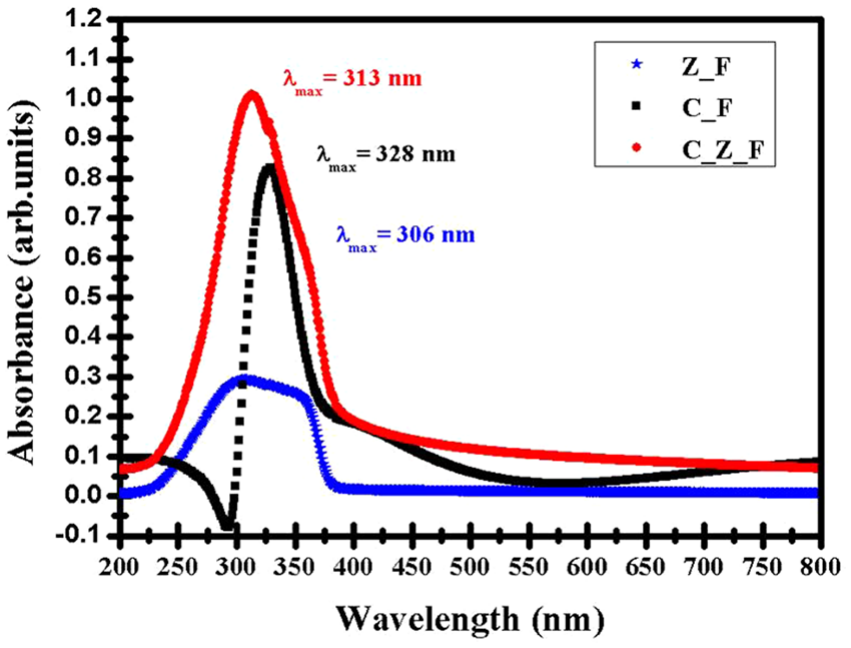
Room temperature UV-Vis absorption spectra of Z_F, C_F and C_Z_F (thickness ~120 ± 10 nm) on quartz substrates.

The optical properties of the as-deposited samples (CeO_2_ film (C_F), Z_F, C_Z_F, Z_NS and C_Z_NS) were investigated by spectroscopic measurements. As mentioned above, the optical absorbance spectra of the films deposited on quartz substrates are recorded in the wavelength range from 200 to 800 nm. Typical absorbance curves for the films grown on quartz are shown in Fig. 5. The influence of adding a CeO_2_ film on top of the ZnO film is clearly observed in these absorbance spectra. The C_Z_F have high absorption in the UV and visible regions followed by a fall-off in the absorption at wavelengths greater than approximately 380 nm. Both pure C_F and Z_F (with approximately the same film thickness of 120 ± 10 nm) have lower absorbance in the visible region and the absorbance spectra of the composite C_Z_F seems to be due to the joint effects of the two constituent oxides. Adding CeO_2_ to the ZnO films clearly increases the absorption in the UV spectral region.

**Figure 6:**
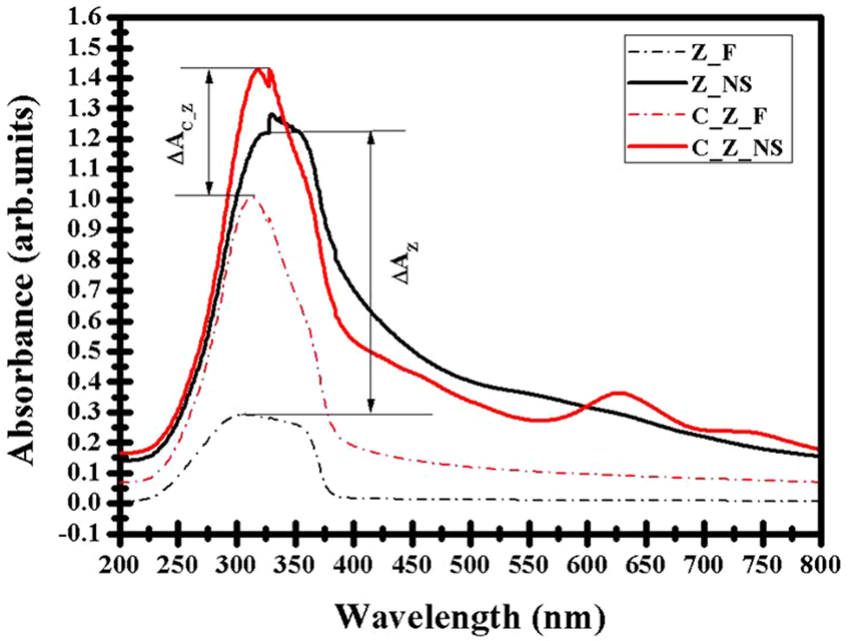
Room temperature UV-Vis absorption spectra of Z_F, Z_NS C_Z_F and C_Z_NS samples, under normal incidence, with a clear indication of an increase in the absorption (ΔA) as a result of the nanoshell structure.

Figure 6 shows the UV-Vis absorption spectra of the pure Z_F, C_Z_F, Z_NS and C_Z_NS samples, with the same physical thicknesses of the two materials in both the film and nanoshell morphologies. All samples are annealed at 500 °C in air for 30 minutes in order to completely eliminate the PS spheres from the nanoshell samples and to enable a direct comparison between the nanoshells and the films. Other Z_F, C_Z_F, Z_NS and C_Z_NS samples were also annealed at 800 °C to crystallise the materials; however no significant differences in absorption, compared to samples annealed at 500 °C, are seen in these samples. It can be clearly seen in Fig. 6 that pure Z_NS and C_Z_NS samples exhibit a much higher UV light absorption level of ~3 times and 1½ times the comparable thin film absorption, respectively. This confirms that a significant enhancement in the UV light absorption is achieved by the engineered spherical nanoshells, for identical sample material thicknesses; hence the geometry of the structure dramatically improves the absorption. It is important to note that the discontinuity at ~330 nm is due to an instrumental artefact (change in grating response) and it is detected in most of our UV-Vis absorption spectra. The samples with the nanoshell morphologies also show distinctly higher apparent absorption in the visible region, compared to equivalent thickness samples with thin film morphologies. We believe that this is due to the effects of increased light scattering and diffraction of energy out of the incident beam, due to the ordered spherical nanoshells structure, which has a periodicity of similar order (600 nm) to visible light wavelengths.

**Figure 7:**
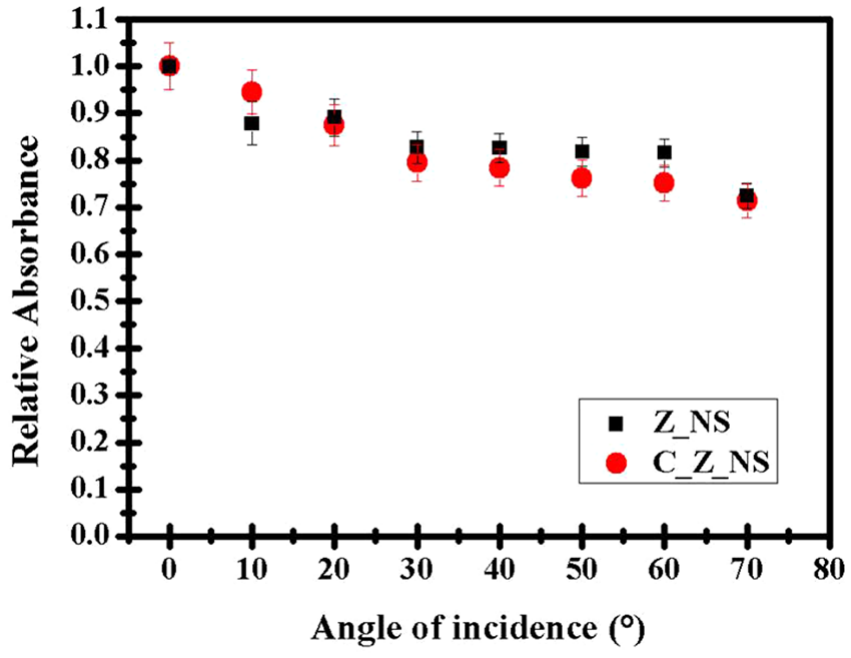
Integrated relative absorbance for different incidence angles relative to normal incidence.

The absorbance enhancement is also relatively insensitive to the angle of incidence, as shown in Fig. 7. Spectrally integrated over the wavelength range of 200–800 nm, the relative absorbance enhancement shows a maximum variation of less than 30% between values measured at normal incidence and at an incidence angle of 70° from the normal for both the C_Z_NS and Z_NS samples, compared to the relevant thin film samples. The relative absorbance enhancement at an angle *i* is calculated using the following formula:

1 $${\rm{R}}{\rm{e}}{\rm{l}}{\rm{a}}{\rm{t}}{\rm{i}}{\rm{v}}{\rm{e}}\,{\rm{A}}{\rm{b}}{\rm{s}}{\rm{o}}{\rm{r}}{\rm{b}}{\rm{a}}{\rm{n}}{\rm{c}}{\rm{e}}\,{\rm{E}}{\rm{n}}{\rm{h}}{\rm{a}}{\rm{n}}{\rm{c}}{\rm{e}}{\rm{m}}{\rm{e}}{\rm{n}}{\rm{t}}\,(i)=\frac{{I}_{A{\rm{\_}}i}}{{I}_{A{\rm{\_}}{0}^{{}^{\circ }}}}$$

*I*_*A_*0°_ and *I*_*A_i*_ are the spectrally integrated absorbance enhancements at normal incidence and an incidence angle of *i* from the normal, respectively. The spectrally integrated absorbance enhancement at all incident angles was determined using:

2 $$\,{I}_{A\_i}={I}_{{A}_{i}}(NS)-{I}_{{A}_{i}}(F)$$

where ${I}_{{A}_{i}}(NS)$ and ${I}_{{A}_{i}}(F)$ are the spectrally integrated absorbances for the nanoshell and thin film samples (at an incidence angle of *﻿i*) with the same physical thicknesses of the materials in both the film and nanoshell morphologies.

## Conclusions

In this paper, we have demonstrated a simple and reproducible method to fabricate ZnO and CeO_2_-coated ZnO thin films and functional nanoshell nanostructures on Si (100) and quartz substrates. The ZnO films and nanostructures were grown by a facile drop coating method using zinc acetate in anhydrous ethanol solution as a starting material while the CeO_2_ deposits were produced by a pulsed DC magnetron sputtering method. XRD, SEM and SIMS measurements were used to confirm the structural, morphological and compositional properties of the deposited materials. In particular XRD data indicated the poorly crystalline nature of the as-deposited ZnO and CeO_2_ nanostructures and showed that the crystalline quality improved after post-deposition annealing at higher temperatures. SEM images showed the successful engineering of the spherical nanoshell structures with a clear indication of the central voids. SIMS analysis of the chemical composition showed the presence of the Ce^+^, CeO^+^, CeO_2_^+^, Zn^+^ and ZnO^+^ ionic species in the various relevant samples as well as the three different Zn isotopes (^64^Zn, ^66^Zn and ^68^Zn), and depth profiling showed the location of the ZnO/CeO_2_ interface in relevant samples. UV and visible light absorption was very significantly enhanced through the engineering of spherical nanoshells on a PS monolayer template, most likely due to the whispering gallery modes in such nanoshell cavities, as well as the addition of the CeO_2_ layer. Our results and analysis clearly show that key materials properties such as the UV and visible light absorption can be significantly enhanced by nanostructure engineering of the deposits to create spherical nanoshell cavities. These results may prove very useful in terms of enabling future materials and device developments, with the aim of controlling key deposit parameters for technologically important applications, in particular in the areas of solar-thermal fuel generation and catalysis, where the combination of the nanostructure engineering possible with ZnO and the oxygen storage and variable stoichiometry properties of CeO_2_ provides a unique set of advantageous deposit properties.

## Electronic supplementary material

Enhanced Optical Properties of ZnO and CeO2-coated ZnO Nanostructures Achieved Via Spherical Nanoshells Growth On A Polystyrene Template
