# Supplementary material for: Enhanced Optical Properties of ZnO and CeO2-coated ZnO Nanostructures Achieved Via Spherical Nanoshells Growth On A Polystyrene Template
Source: Sci Rep. 2017 Jun 16;7:3737. doi: 10.1038/s41598-017-03905-4 (PMC5473917; doi:10.1038/s41598-017-03905-4)
Supplement: Supplementary file 1 — Enhanced Optical Properties of ZnO and CeO2-coated ZnO Nanostructures Achieved Via Spherical Nanoshells Growth On A Polystyrene Template [file 41598_2017_3905_MOESM1_ESM.pdf]

Enhanced Optical Properties of ZnO and CeO_2-coated ZnO Nanostructures Achieved Via Spherical Nanoshells Growth On A Polystyrene Template

Asmaa Eltayeb[a,*], Stephen Daniels[a] and Enda McGlynn[b]

[a] *School of Electronic Engineering, National Centre for Plasma Science and Technology, Dublin City University, Glasnevin, Dublin 9, Ireland. Email: stephen.daniels@dcu.ie*

[b] *School of Physical Sciences, National Centre for Plasma Science and Technology, Dublin City University, Glasnevin, Dublin 9, Ireland. Email: enda.mcglynn@dcu.ie*

* *Contact Author: asmaa.eltayeb2@mail.dcu.ie*

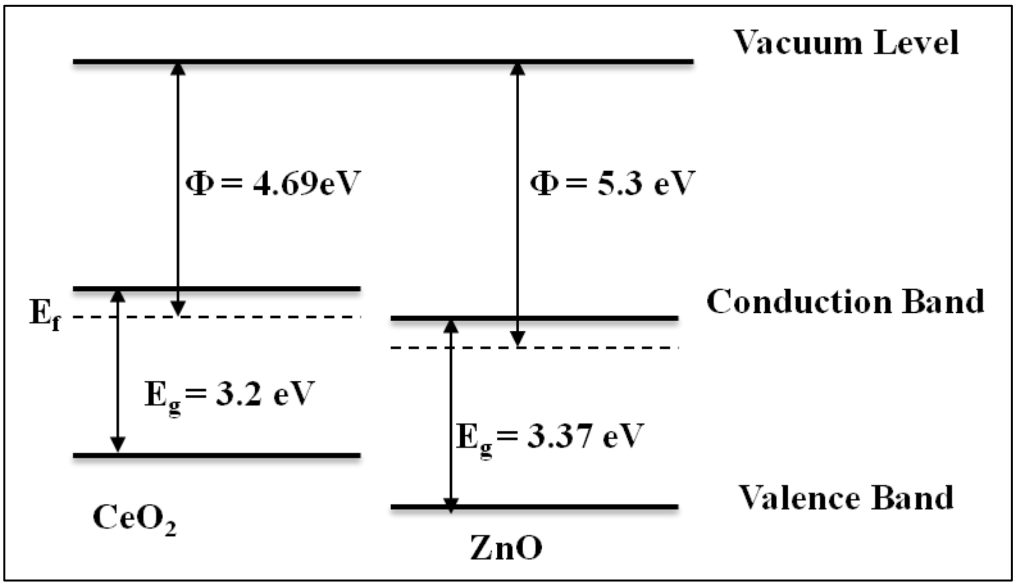

Figure I: Schematic of the energy band alignment between ZnO and CeO_2 [1]

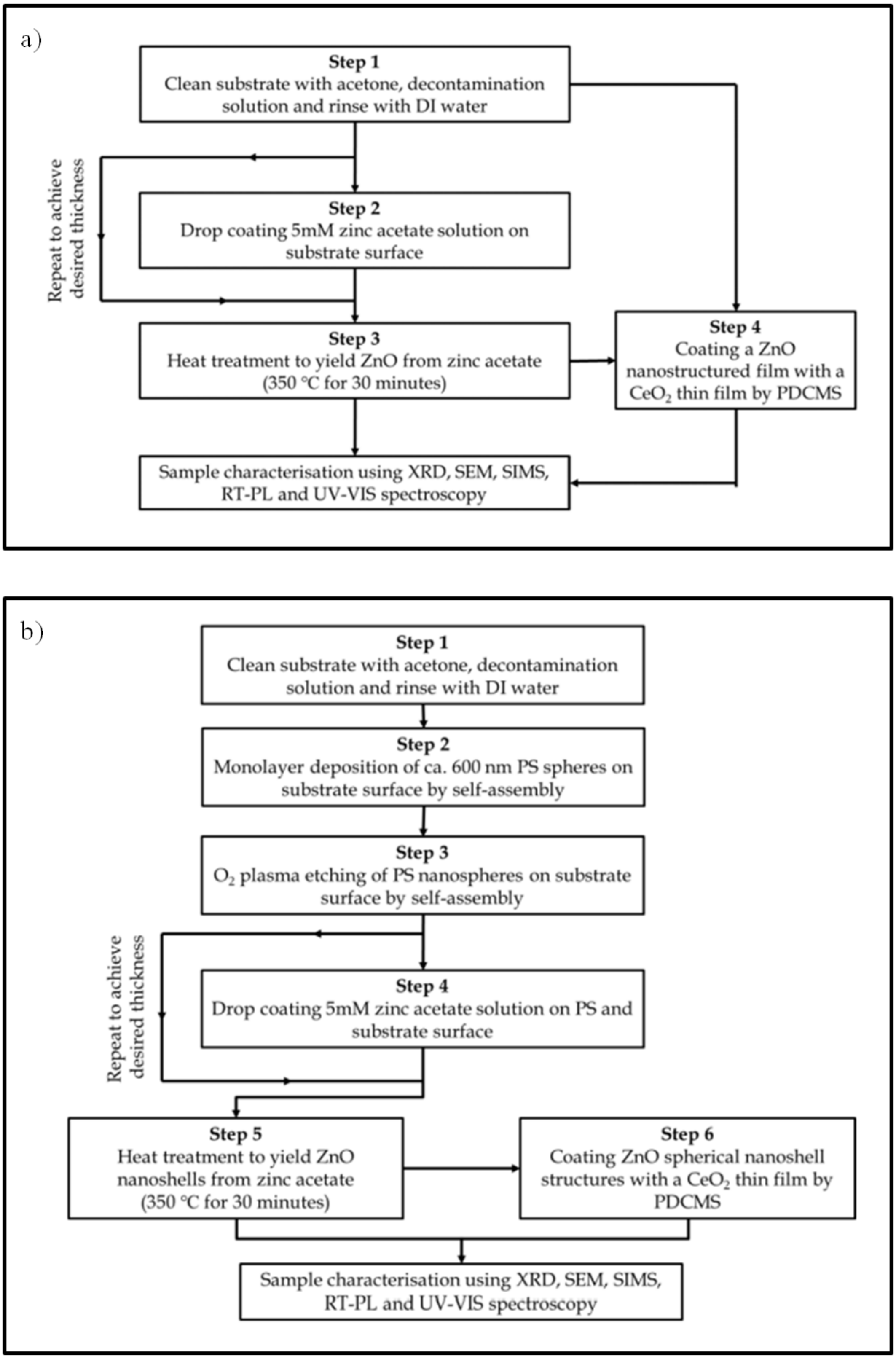

Figure II: Flow chart of the overall process in the fabrication of CeO_2, ZnO and CeO_2-coated ZnO a) films and b) spherical nanoshell structures by zinc acetate drop coating and pulsed DC magnetron sputtering deposition methods on bare substrates.

S1. XRD MEASUREMENTS:

XRD patterns of the Z_F and Z_NS, as-deposited and annealed at 500 °C and 800 °C in air for 30 minutes are shown in Figure III. The XRD scans of all the Z_F and Z_NS show a dominant peak at 34.4°, corresponding to the (002) plane of the ZnO wurtzite phase (JCPDS card number 36-1451). This indicates the ZnO nanostructures are highly textured with their c-axis normal to the substrate [2,3], which indicates that the nanocrystals in the seed layer remain textured normal to the substrate in both the film and nanoshell samples, and indicate the dominant effect of inter-nanocrystal basal plane interactions during deposition. The annealed Z_F also show two small peaks at 31.7° and 36.2° which correspond to the ZnO (100) and (101) planes, respectively.

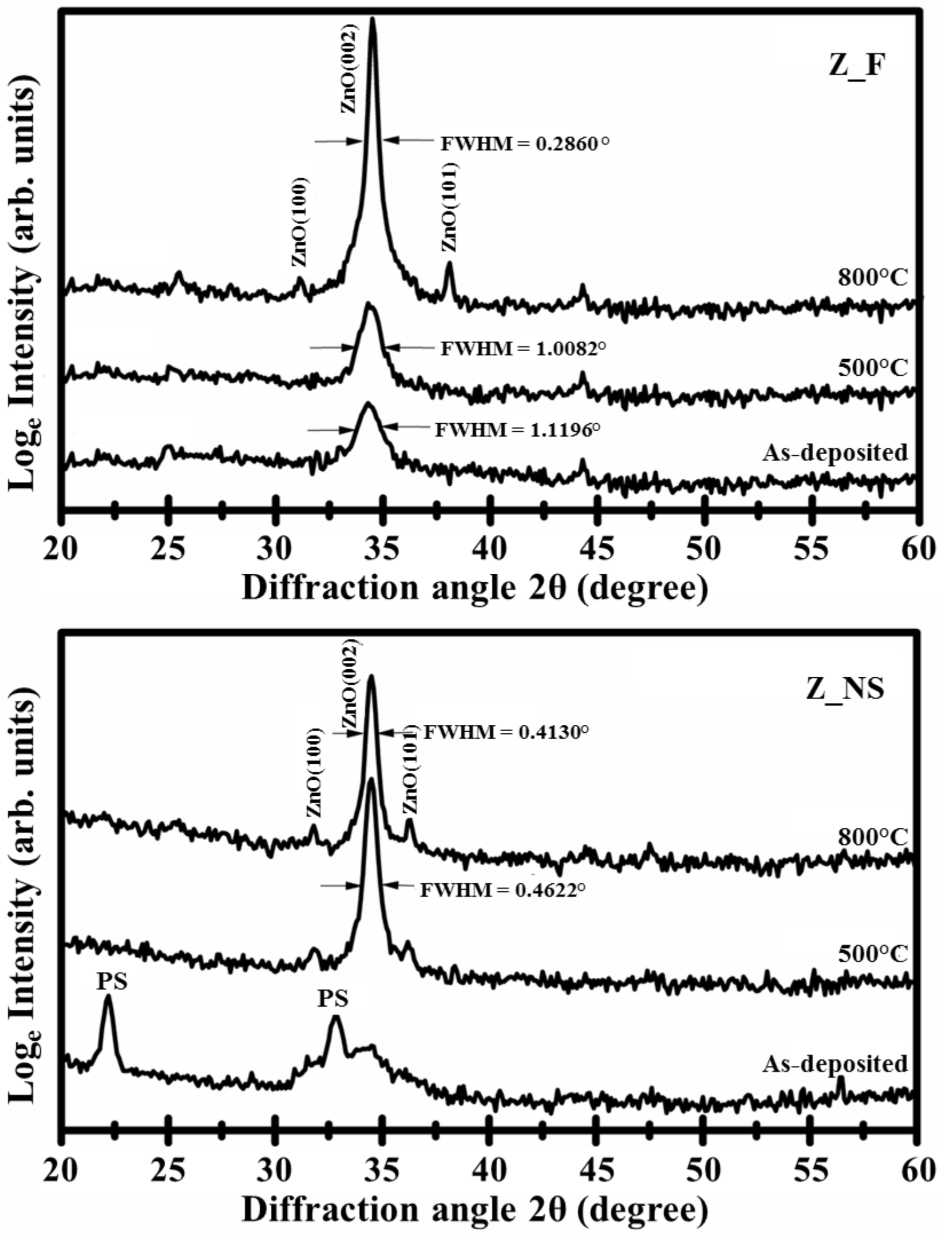

Figure III: XRD θ/2θ scan (locked coupled) of Z_F (top) and Z_NS structures (bottom) grown on Si (100) substrates. The samples are as-deposited and annealed at 500 °C and 800 °C for 30 minutes. The dominant XRD peaks located at 34.4° correspond to the ZnO (002) reflection.

In the as-deposited Z_NS sample, two polystyrene ($(C_8H_8)_n$) related peaks are detected. These peaks are only present on the as-deposited nanoshells which are only annealed at 350 °C for 30 minutes during growth to decompose the zinc salt into zinc oxide,

which is less than the evaporation temperature needed to remove the PS spheres. A broad peak at 34.4° is detected indicating an intermediate nanocrystalline/ poorly crystalline ZnO deposit. It is important to note that the samples annealed at 500 °C and 800 °C for 30 minutes have no remaining peaks associated with PS nanospheres. In these annealed nanoshells samples we once again see the dominant peak at 34.4°, corresponding to the (002) plane of the ZnO wurtzite phase, as well as two small peaks at 31.7° and 36.2° which correspond to the ZnO (100) and (101) planes, respectively.

Similarly, the XRD patterns of the C_Z_F and C_Z_NS, as-deposited and annealed at 500 °C and 800 °C in air for 30 minutes, are shown in Figure IV. The XRD of the as-deposited films is similar to that of the Z_F as no peaks associated with the CeO_2 are detected. This indicates the poorly crystalline nature of the as-deposited CeO_2 films. However, as the C_Z_F are annealed at 500°C and 800 °C, more peaks are detected. These peaks are identified as either ZnO with wurtzite hexagonal structure ($2\theta = 34.4°$) or CeO_2 (JCPDS card number 34-0394) with cubic structure ($2\theta = 28.5°$, 33.0°, 47.4°, 56.3°, and 58.9°). Again, this indicates highly textured ZnO films with their c-axis normal to the substrate. No peaks assignable to Ce^{III} compounds, such as Ce_2O_3 and $Ce(OH)_3$ are seen in the pattern, however a small peak indicated (*) is detected in the 500°C C_Z_NS which its origin has not yet been identified. Furthermore, two small peaks at 31.7° and 36.2° assigned to ZnO(100) and ZnO(101), respectively, are detected in the 800°C CeO_2-coated ZnO films. There are no substantial differences between the XRD patterns of the C_Z_F samples compared to those of the C_Z_NS structure samples shown below, and the 800 °C annealed samples are virtually identical. The crystallinity of the CeO_2 and ZnO phases after the 500 °C anneal is much higher for the films compared to the nanoshell structures, based on the FWHM values of the dominant peaks. However, it is important to note that the XRD pattern of the as-deposited C_Z_NS sample displays all the peaks associated with both the CeO_2 and ZnO phases. The origin of this difference in crystallisation behaviour is not yet clear.

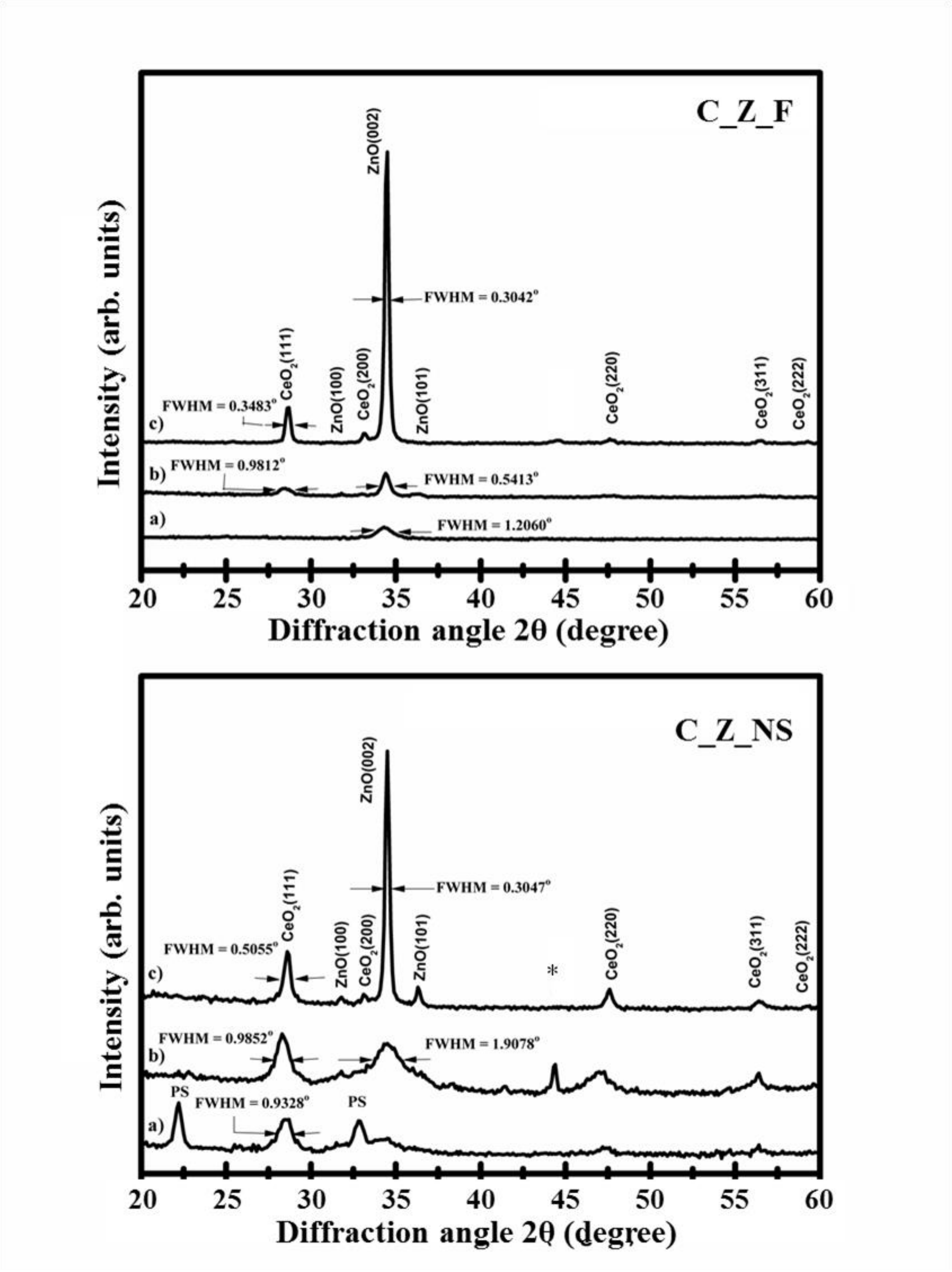

Figure IV: XRD θ/2θ scan (locked coupled) of C_Z_F (top) and C_Z_NS structures (bottom) grown on Si (100) substrates. The samples are a) as-deposited and annealed at b) 500 °C and c) 800 °C for 30 minutes. The dominant XRD peaks located at 34.4° and 28.5° correspond to the ZnO (002) and CeO_2 (111) reflections, respectively.

The reflected x-ray intensity and reflection peak FWHM of the CeO_2 (111) and ZnO (002) XRD peaks are used as an indicator of the crystallinity quality of the CeO_2 and ZnO deposits. As can be seen in Figure III and IV, an increase in the annealing temperature resulted in an increased XRD reflection intensity and a decrease of the FWHM of the CeO_2 (111) and ZnO (002) XRD peaks. In the 500 °C annealed CeO_2-coated ZnO films, for example, FWHM values of 0.9852° and 1.9078° are measured for CeO_2 (111) and ZnO (002) XRD peaks, respectively. These values decreased to 0.5055° and 0.3047° for the samples annealed at 800 °C. This trend is the same for all the other ZnO and CeO_2-coated ZnO samples (see Table I); indicating that the crystalline quality of the samples is systematically improved as a result of annealing.

Table I: Summary of the FWHM values of the ZnO (002) XRD peaks observed for the various samples.

	FWHM (°)		
**Sample**	**As-deposited**	**Annealing Temperature (°C)**	
		**500**	**800**
Z_F	1.1196	1.0082	0.2860
Z_NS	-	0.4622	0.4130
C_Z_F	1.2060	0.5413	0.3042
C_Z_NS	-	1.9078	0.3047

S2. CHEMICAL COMPOSITION MEASUREMENTS:

To provide information on the chemical composition and impurity content in the samples and confirm the composition of C_Z_F, SIMS measurements were undertaken at different locations throughout the deposit. Figure V shows the SIMS spectra of the C_Z_F in the mass region from 60 to 200 amu, at the boundary where the two materials meet. As reported previously [4], sputtered CeO_2 SIMS spectra showed an intense secondary ion peak of CeO^+ and two lower intensity peaks of Ce^+ and CeO_2^+. This can be clearly observed in Figure V (spectrum 370). As more scans are performed and the probing depth increased due to surface sputtering by the Ga ion beam, Zn+ and ZnO^+ peaks start to appear and their intensity increased with the increase in the number of scans. Three different Zn isotopes are observed for the Zn ions, ^{64}Zn, ^{66}Zn and ^{68}Zn, with the highest intensity observed for the ^{64}Zn isotope, consistent with the natural isotopic distribution. SIMS spectra from Z_F displayed similar peaks to that of spectrum 490 in Figure V, with a constant intensity for all the Zn and ZnO peaks throughout the sample. Note that (i) the intensity of the peaks depends significantly on

the sputtering rate of the components during the SIMS scan and (ii) that each spectrum is taken over a 10.2 second sputtering period i.e. spectrum 370 is recorded after 62.9 minutes (3774 seconds) total sputtering time.

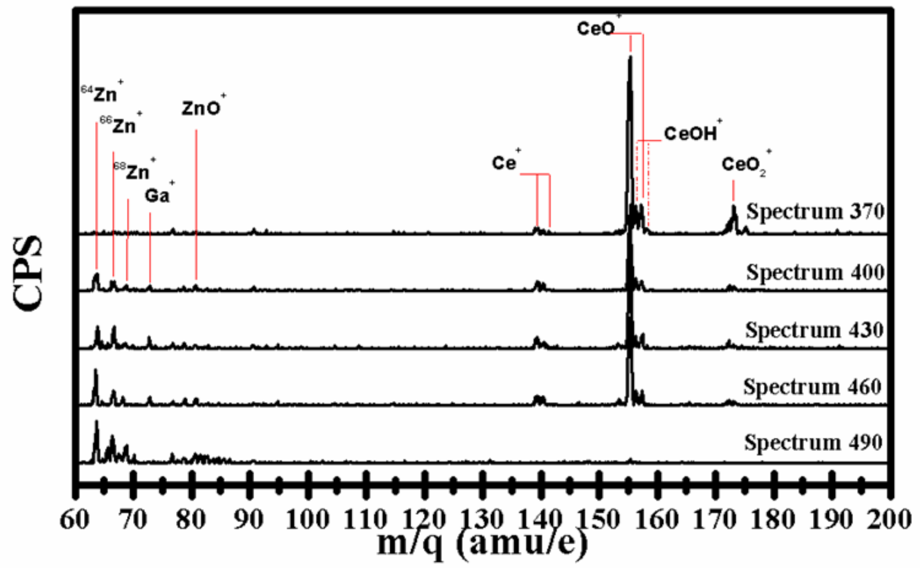

Figure V: SIMS spectra of positive secondary ions at the boundary of the C_Z_F and C_Z_NS samples. Increasing spectrum number indicates increased overall sputtering time, as discussed in the main text.

The SIMS depth profiling data of the relative secondary ion emission yields ($^{64}Zn^+$, Ce^+, CeO^+ and CeO_2^+) as a function of depth at the boundary of the CeO_2-coated ZnO composite layers grown on Si(100) substrate is presented in Figure VI. Since the sputtering rate of the different ions varies from one element or compound to another, the count intensity or observed signal strengths are not directly inter-comparable in terms of chemical concentrations. Multiple scans were performed at the same location on the C_Z_F. An almost uniform signal level is seen for the Ce^+, CeO^+ and CeO_2^+ ion profiles throughout the CeO_2 layer at depth numbers between 1 and 310. As the exposure time increased, the effect of surface sputtering also increased resulting in a deeper penetration through the films. Therefore, a significant decrease in magnitude of the CeO^+ count intensity with respect to the Ce^+ and $CeO_2^{+,}$ and an increase of the $^{64}Zn^+$ count intensity is seen, indicating the location of the ZnO/CeO_2 interface. Although a significant reduction of CeO^+ intensity is recorded as the Zn signal begins to increase, it remains the signal with the highest intensity. The SIMS data

show clear evidence of an abrupt interface between the ZnO and CeO_2 materials. The difference in intensities is due to to the sputtering rates of the various elements and components and therefore, lower intensities were recorded for Ce^+ and CeO_2^+.

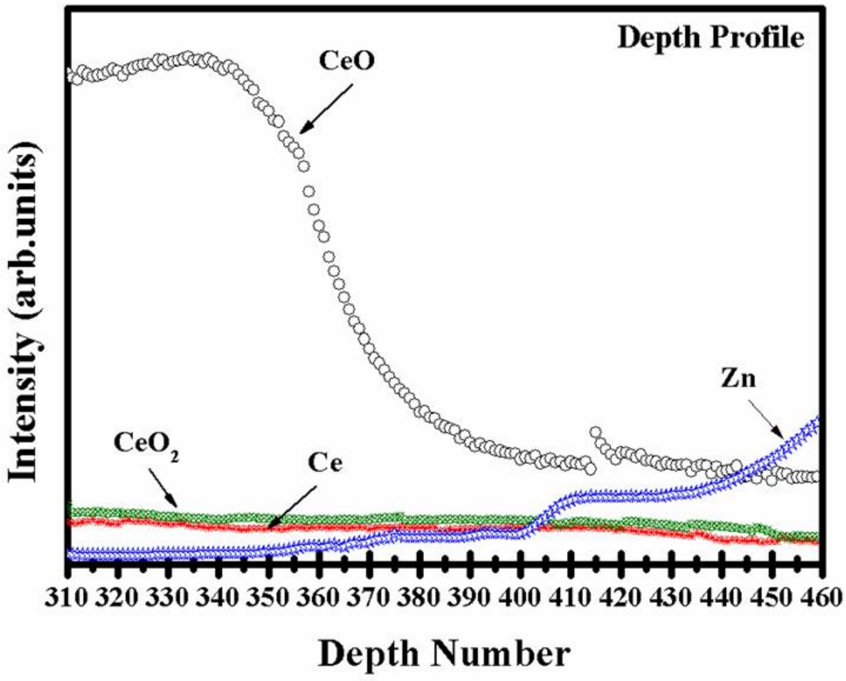

Figure VI: Relative secondary ion emission yields for Zn, Ce, CeO and CeO_2 positive ions as a function of depth at the boundary of the composite C_Z_F layer grown on a Si (100) substrate.

References

1 Warule, S. S. *et al.* Decoration of CdS nanoparticles on 3D self-assembled ZnO nanorods: a single-step process with enhanced field emission behaviour. *CrystEngComm* **17**, 140-148 (2015).

2 Byrne, D. *The growth and characterisation of ordered arrays of zinc oxide nanostructures and optical studies of defects in zinc oxide* PhD thesis, Dublin City University, (2012).

3 McCarthy, E., Garry, S., Byrne, D., McGlynn, E. & Mosnier, J.-P. Field emission in ordered arrays of ZnO nanowires prepared by nanosphere lithography and extended Fowler-Nordheim analyses. *Journal of Applied Physics* **110**, 124324 (2011).

4 Eltayeb, A. *et al.* Control and enhancement of the oxygen storage capacity of ceria films by variation of the deposition gas atmosphere during pulsed DC magnetron sputtering. *Journal of Power Sources* **279**, 94-99 (2015).
